# Associations between obesity, smoking and lymph node status at breast cancer diagnosis in the Prostate, Lung, Colorectal and Ovarian (PLCO) Cancer Screening Trial

**DOI:** 10.1371/journal.pone.0202291

**Published:** 2018-08-29

**Authors:** Amelia Smith, Maeve Mullooly, Laura Murphy, Thomas Ian Barron, Kathleen Bennett

**Affiliations:** 1 Dept. Pharmacology and Therapeutics, Trinity Centre for Health Sciences, St. James’ Hospital, Dublin, Ireland; 2 Division of Cancer Epidemiology and Genetics, National Cancer Institute, Rockville, Maryland, United States of America; 3 Cancer Prevention Fellowship Program, Division of Cancer Prevention, National Cancer Institute, Bethesda, Maryland, United States of America; 4 Division of Population Health Sciences, Royal College of Surgeons in Ireland, Dublin, Ireland; 5 Department of Epidemiology, Johns Hopkins Bloomberg School of Public Health, Baltimore, Maryland, United States of America; University of Cincinnati College of Medicine, UNITED STATES

## Abstract

**Introduction:**

There is evidence suggesting that smoking and obesity prior to a breast cancer diagnosis is associated with poorer outcomes. In this study, we investigate the associations between smoking and obesity prior to a breast cancer diagnosis and the presence of lymph node metastases at diagnosis.

**Methods:**

Women with stage I-III breast cancer (n = 3,304) were identified from the National Cancer Institute’s Prostate, Lung, Colorectal and Ovarian Cancer Screening Trial. Univariable and multivariable log-binomial models were used to estimate relative risks (RR) and 95% confidence intervals (CIs) for associations between lymph node positive breast cancer and; i) smoking, and ii) obesity prior to diagnosis.

**Results:**

Pre-diagnostic smoking/obesity was not associated with lymph node metastasis at diagnosis in multivariable analyses; (RR 0.82, 95%CI 0.61, 1.10) and (RR 0.95, 95% CI 0.81, 1.12), respectively.

**Conclusion:**

Obesity and smoking information was recorded a number of years prior to breast cancer diagnosis, therefore these findings should to be replicated in a larger cohort of women, with more detailed smoking and obesity information.

## Introduction

Obesity and smoking in women prior to a breast cancer diagnosis has been shown to be associated with disease recurrence and reduced breast cancer-specific survival [1,2]. It is well established that cigarette smoking is linked to cancers such as lung and stomach, and although cigarette smoke contains mammary carcinogens, the link to breast cancer is less certain [3]. In a meta-analysis of 10 studies investigating the association between smoking at diagnosis and breast cancer specific mortality, current smokers had a statistically significant 33% increase in breast cancer specific mortality (hazard ratio (HR) 1.33, 95% confidence intervals (CI) 1.12, 1.58) compared to never smokers [1].

Obesity at breast cancer diagnosis has been linked to aggressive tumours and an overall worse prognosis[2]. In a meta-analysis of 22 studies investigating pre-diagnostic body mass index (BMI) and breast cancer specific-mortality, obese women were 35% more likely to die of their breast cancer, when compared to women with a normal pre-diagnostic BMI (Relative risk (RR) 1.35, 95% CI 1.24, 1.47)[4]. Overweight and obese women have increased levels of; insulin-like growth factor (IGF), TNF-α, IL-6, and VEGF, which can promote angiogenesis, tumour growth, metastasis and cell survival [5]. Both smoking and obesity can induce a pro-inflammatory environment[6]. Inflammation has been shown to possess a variety of tumorigenic properties; enhancing cancer cell survival, promoting angiogenesis, and promoting metastatic dissemination [7]. Lymph node (LN) status has been shown to be an important prognostic factor in several breast cancer subtypes, and can influence important clinical decisions such as the potential therapeutic options, subsequent quality of life and medical costs [8]. Additionally, predictive models have been developed for to predict LN metastasis but these are not well integrated into clinical practice and often do not include lifestyle exposures [9,10]. As such, it is vital to determine the possible effects of lifestyle factors on LN status at breast cancer diagnosis. Therefore, we aimed to examine the associations between; i) pre-diagnostic obesity; and ii) pre-diagnostic smoking; and the presence of LN metastasis at breast cancer diagnosis among women in the US PLCO population[11]. In the analyses of pre-diagnostic smoking and presence of LN metastasis, we also investigated the presence of effect modification by pre-diagnostic obesity.

## Methods

### Setting and data collection

This study was carried out using data from the Prostate, Lung, Colorectal and Ovarian (PLCO) Cancer Screening Trial. The PLCO trial was a multicentre, randomized clinical trial designed to evaluate the effect of screening for prostate, lung, colorectal, and ovarian cancer on cancer-specific mortality in over 150,000 individuals in the United States (US). The PLCO study protocol was approved by the Institutional Review Board of the National Cancer Institute and all participating institutions. All participants provided written informed consent upon enrolment. PLCO has the following five ClinicalTrials.org registration numbers: NCT00002540 (Prostate), NCT01696968 (Lung), NCT01696981 (Colorectal), NCT01696994 (Ovarian), and NCT00339495 (EEMS), and details of the methodology of the PLCO trial have been described previously [12]. Participants completed a baseline questionnaire (BQ) at trial entry and an annual update questionnaire requesting details of cancer diagnoses within the previous year. All self-reported cancers were confirmed through medical record review by trained abstractors.

### Participants

For this study, we included post-menopausal women who completed the PLCO BQ that were diagnosed with stage I–III breast cancer between 1993 and 2009 (n = 3,304 women). Women were excluded if they were missing information concerning smoking status, BMI, LN status or had a previous invasive cancer diagnosis. Women were followed until time of death, loss to follow-up, December 31st 2009, or a maximum of 13 years; whichever occurred earliest. Information regarding smoking status and BMI were obtained from the BQ. Smoking status was categorised as current, former, or never smoker. BMI was categorised as; <18.5 kg/m^2^ (underweight), 18.5–24.9 kg/m^2^ (normal weight), 25–29.9 kg/m^2^ (overweight) and > = 30 kg/m^2^ (obese). The median time between completion of the BQ and diagnosis of breast cancer was 5.9 years (min; 6 days, max; 15.9 years).

### Outcomes and covariates

Women were identified as LN positive if they had a pathologic LN status of pN1, pN2, or pN3 (see S1 Table). Based on prior literature, we included important predictors of LN status in multivariable models [13]. For example, pre-diagnostic exposure to non-steroidal anti-inflammatory drugs (NSAIDs) is linked to reduced likelihood of presenting with lymph node positive breast cancer at diagnosis [14]. Tumour characteristics such as grade and hormone receptor status are also liked to lymph node status at breast cancer diagnosis [15,16]. Information regarding the following breast tumour characteristics was obtained from medical records: tumour stage (I, IIA, IIB, IIIA, or IIIB); tumour grade (1, 2, or 3/4); tumour size (cm), tumour morphology (lobular, tubular, ductal, other), estrogen receptor (ER) status (positive, negative, unspecified), progesterone receptor (PR) status (positive, negative, unspecified), and human epidermal growth factor receptor 2 (HER2) status (positive, negative, unspecified). Additional data obtained from the BQ included; smoking status (current, former, never), use of NSAIDs (ibuprofen and/or aspirin; yes or no). An indicator of comorbidity (score 0–3) was developed based on reporting of 1 or more of the following conditions at baseline: heart attack, diabetes, hepatitis, stroke, arthritis.

### Statistical analysis

LN status was tabulated and differences in the proportion of women with/without LN metastasis were compared for obesity and smoking levels using univariate Poisson regression. Univariable and multivariable log-binomial models were used to estimate relative risks (RR) and 95% confidence intervals (CI) for associations between LN positive breast cancer and;

1. pre-diagnostic obesity
2. pre-diagnostic smoking (current smoker at time of BQ)
3. ever pre-diagnostic smoking (current and former smokers at BQ)

The covariates described were identified for inclusion in the multivariable model based on prior knowledge of clinical and demographic predictors of positive lymph node status. In analyses ii) and iii), the presence of effect modification by obesity was assessed by inclusion of an interaction term in the multivariable model.

We also carried out a *post-hoc* analysis, whereby the association between LN positive breast cancer and i) pre-diagnostic obesity, ii) pre-diagnostic smoking (current smoker at time of BQ), and iii) ever pre-diagnostic smoking (current and former smokers at BQ) was stratified by time from BQ to breast cancer diagnosis (less than 3 years, 3 years or greater).

## Results

### Participant characteristics

**Fig 1:**
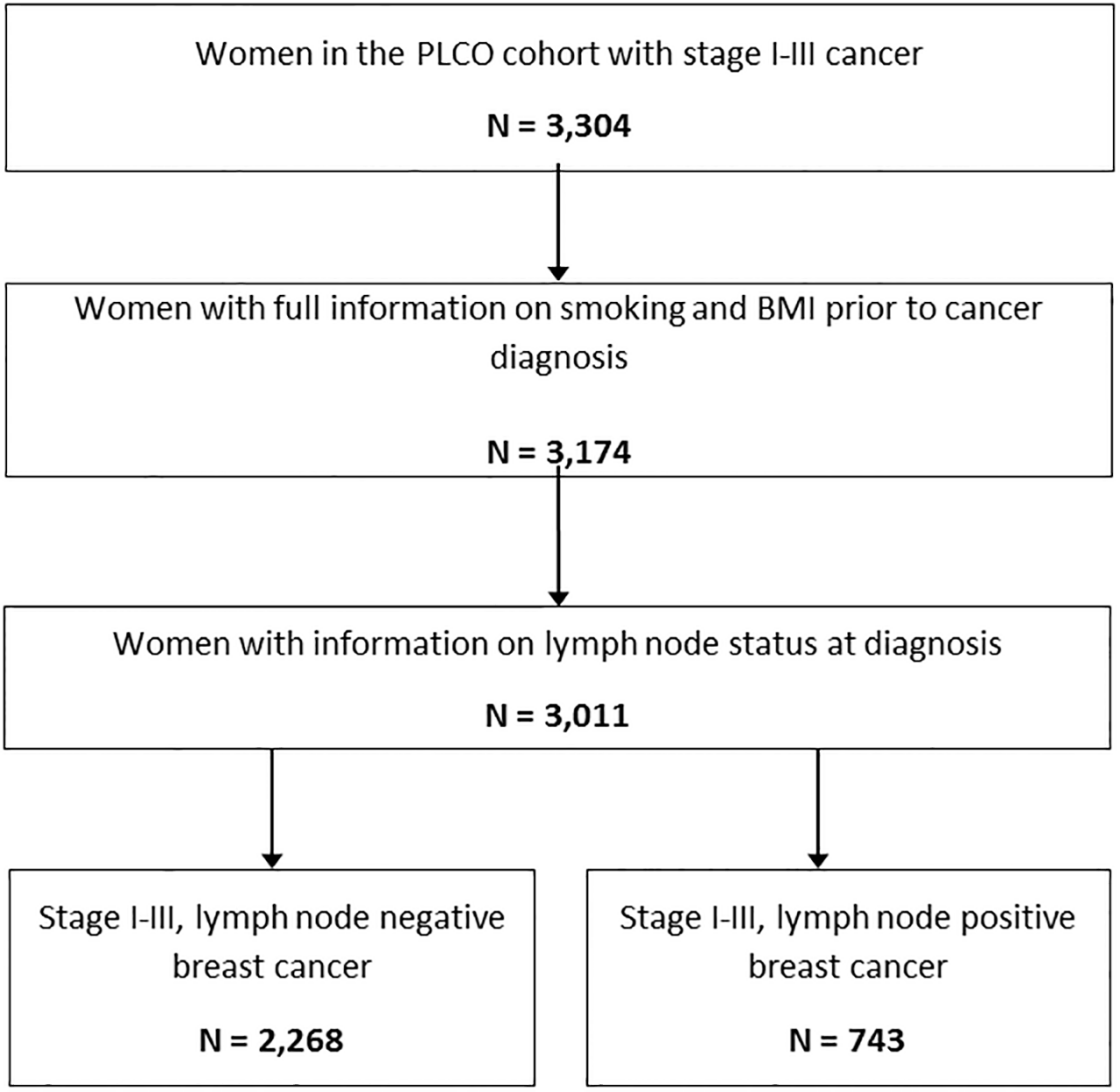
Flow diagram showing study cohort selection.

We identified 3,304 women with stage I-III breast cancer. Of these, 3,011 (91%) women had complete information on pre-diagnostic smoking status, pre-diagnostic obesity status and lymph node status at diagnosis (Fig 1). The characteristics of these women are presented in Table 1. The median age at diagnosis in the cohort was 67 (interquartile range; 63, 73) and the majority of women were white non-Hispanic. In univariable analysis, women with lymph node positive status (n = 743, 24.7%) were significantly more likely to have larger tumours and a higher tumour stage and grade and were also significantly more likely to be PR and HER2 positive (Table 1).

**Table 1:** Characteristics of women selected for inclusion in study cohort.

Characteristic		LN - *N* = 2,268 (75.3%)	LN + *N* = 743 (24.7%)
Age in years	Median (IQR)	68 (63, 73)	67 (62, 71)
Age Categories—(%)*	55–59	188 (8.3)	83 (11.2)
	60–64	511 (22.5)	193 (26.0)
	65–69	643 (28.6)	209 (28.1)
	70–74	515 (22.7)	144 (19.4)
	75–79	300 (13.2)	75 (10.1)
	80–84	98 (4.3)	38 (5.1)
	85–89	13 (0.4)	1 (0.1)
Comorbidity score	0	1100 (48.5)	363 (48.9)
	1	984 (43.4)	316 (42.5)
	2	164 (7.2)	54 (7.3)
	3	20 (0.9)	10 (1.4)
Smoking—(%)	Current	1245 (54.9)	429 (57.7)
	Past	189 (8.3)	49 (6.6)
	Never	834 (36.8)	265 (35.7)
BMI kg/m2—(%)	<18.5	19 (0.8)	3 (0.4)
	18.5–25	869 (38.3)	282 (38.0)
	25–30	819 (36.1)	261 (35.1)
	>30	561 (24.7)	197 (26.5)
Tumour Morphology (%)*	Lobular	258 (11.4)	114 (15.3)
	Tubular	62 (2.7)	4 (0.5)
	Ductal	1701 (75.0)	553 (74.4)
	Other	247 (10.9)	72 (9.7)
NSAID—(%)	Yes	1320 (58.2)	439 (59.1)
	No	948 (41.8)	304 (40.9)
Tumour size—(%)*	<2cm	1906 (84.0)	377 (50.7)
	2cm-5cm	341 (15.0)	301 (40.5)
	>5cm	20 (0.9)	57 (7.7)
	Missing	1 (0.1)	8 (1.1)
Tumour stage—(%)*	I	1905 (84.0)	0 (0.0)
	IIa	336 (14.8)	359 (48.3)
	IIb	18 (0.8)	272 (36.6)
	IIIa	0 (0.0)	79 (10.6)
	IIIb-c	9 (0.4)	33 (4.4)
Tumour grade—(%)*	I	715 (31.5)	114 (15.3)
	II	944 (41.6)	336 (45.2)
	III	447 (19.7)	252 (33.9)
	IV	7 (0.3)	1 (0.1)
	Not determined	155 (6.8)	40 (5.4)
ER—(%)	Negative	306 (13.5)	120 (16.2)
	Positive	1813 (79.9)	585 (78.7)
	Unspecified	149 (6.6)	38 (5.1)
PR—(%)*	Negative	492 (21.7)	189 (25.4)
	Positive	1605 (70.8)	512 (68.9)
	Unspecified	171 (7.5)	42 (5.7)
HER2—(%)*	Negative	1342 (59.2)	422 (56.8)
	Positive	248 (10.9)	137 (18.4)
	Unspecified	678 (29.9)	184 (24.8)

Abbreviations: LN = lymph node, IQR = interquartile range, BMI = body mass index, NSAID = non-steroidal anti-inflammatory drug, ER = estrogen receptor, PR = progesterone receptor, HER2 = human epidermal growth factor receptor

*Difference in variable between lymph node status; p <0.05 Poisson regression

### Pre-diagnostic obesity and smoking, and lymph node status at diagnosis

Relative risks for associations between pre-diagnostic smoking, obesity, and lymph node status are presented in Table 2. No significant association was found between pre-diagnostic obesity and lymph node status at diagnosis, in univariable (RR 1.07, 95% CI 0.93, 1.23) or multivariable analyses (RR 0.95, 95% CI 0.81, 1.12) (Table 2).

**Table 2:** Univariable and multivariable RR’s and 95% CI’s for associations between pre-diagnostic obesity, smoking and lymph node positive breast cancer.

				LN Positive Breast Cancer				
	LN-	(%)	LN +	(%)	Univariate RR (95% CI)		Multivariate RR (95% CI) ^A^	
Non-obese	1707	(75.7)	546	(24.3)	Ref	-	Ref	-
Obese pre-diagnosis	561	(74.0)	197	(26.0)	1.07	(0.93, 1.23)	0.95	(0.81, 1.12)
Non-smoker	2079	(75.0)	694	(25.0)	Ref	-	Ref	-
Smoker pre-diagnosis	189	(79.4)	49	(20.6)	0.82	(0.64, 1.06)	0.82	(0.61, 1.10)
*obese							0.98	(0.54, 1.78)
Non-smoker	1245	(74.4)	429	(25.6)	Ref	-	Ref	-
Ever-smoker	1023	(76.5)	314	(23.5)	0.91	(0.80, 1.03)	0.95	(0.83, 1.10)
*obese							0.94	(0.74, 1.19)

^A)^ Adjusted for age at diagnosis, comorbidities, use of NSAIDs, ER/PR/HER2 receptor status, breast cancer stage at diagnosis, breast cancer grade at diagnosis.

No significant association was found between pre-diagnostic smoking and lymph node status at diagnosis, in univariable (RR 0.82, 95% CI 0.64, 1.06) and multivariable adjusted analyses (RR 0.82, 95% CI 0.61, 1.10). In analyses of effect modification by obesity in pre-diagnostic smokers, the multivariable relative risk remained non-significant (RR 0.98, 95%CI 0.54, 1.78).

In analyses whereby current and former smokers were grouped into ‘ever’ smokers, results remained the same; no associations were observed between smoking and lymph node status in univariable (RR 0.91, 95% CI 0.80, 1.03) or multivariable analyses (RR 0.95, 95% CI 0.83, 1.10). In analyses of effect modification by obesity in pre-diagnostic ever smokers, the multivariable relative risk remained non-significant (RR 0.94, 95%CI 0.74, 1.19) (Table 2).

### Pre-diagnostic obesity and smoking, and lymph node status at diagnosis, stratified by time from BQ to breast cancer diagnosis

Relative risks for associations between pre-diagnostic smoking, obesity, and lymph node status, when stratified by time from BQ to breast cancer diagnosis are presented in Table 3. No significant association was found between pre-diagnostic obesity or smoking and lymph node status at diagnosis, when stratified by time from BQ (less than 3 years, 3 years or greater) to breast cancer diagnosis.

**Table 3:** Univariable and multivariable RR’s and 95% CI’s for associations between pre-diagnostic obesity, smoking and lymph node positive breast cancer, by time from BQ to diagnosis.

				LN Positive Breast Cancer				
	LN-	(%)	LN +	(%)	Univariate RR (95% CI)		Multivariate RR (95% CI) ^A^	
**Time since BQ <3 years**								
Non-obese	427	72.5	162	27.5	Ref	-	Ref	-
Obese pre-diagnosis	132	75.0	44	25.0	0.91	0.68, 1.21	0.82	0.58, 1.16
Non-smoker	508	72.5	193	27.5	Ref	-	Ref	-
Smoker pre-diagnosis	51	79.7	13	20.3	0.74	0.45, 1.21	0.76	0.45, 1.35
*obese							0.38	0.05, 2.70
Non-smoker	283	70.8	117	29.2	Ref	-	Ref	-
Ever-smoker	276	75.6	89	24.4	0.83	0.66, 1.06	0.88	0.67, 1.17
*obese							0.66	0.38, 1.16
**Time since BQ > = 3 years**								
Non-obese	1280	76.9	384	23.1	Ref	-	Ref	-
Obese pre-diagnosis	429	73.7	153	26.3	1.14	0.97, 1.34	0.99	0.81, 1.20
Non-smoker	1571	75.8	501	24.2	Ref	-	Ref	-
Smoker pre-diagnosis	138	79.3	36	20.7	0.86	0.63, 1.16	0.84	0.60, 1.19
*obese							1.13	0.60, 2.12
Non-smoker	962	75.5	312	24.5	Ref	-	Ref	-
Ever-smoker	747	76.9	225	23.1	0.95	0.81, 1.10	0.98	0.82, 1.17
*obese							1.01	0.78, 1.33

^A)^ Adjusted for age at diagnosis, comorbidities, use of NSAIDs, ER/PR/HER2 receptor status, breast cancer stage at diagnosis, breast cancer grade at diagnosis.

## Discussion

In this study of 3,011 post-menopausal women with stage I-III breast cancer in the PLCO cohort, pre-diagnostic smoking and/or obesity was not significantly associated with positive lymph node status at diagnosis.

Cigarette smoke has been shown to induce cyclooxygenase (COX-2) expression and increase prostaglandin E2 (PGE2) release[17]. Induction of these inflammatory pathways has been linked to the stimulation of lymphangiogensis in *in-vitro* and *in-vivo* breast cancer models [18]. While no association between pre-diagnostic smoking and LN metastasis was observed in this study, this may have been due to the small sample size and the recording of smoking status may predate breast cancer diagnosis by a number of years.

Obesity is a pro-inflammatory condition [19] and chronic inflammation can lead to induction of tumorigenic pathways[20]. In postmenopausal women the majority of estrogen is produced in the peripheral adipose tissue, and is increased in women who are overweight and obese [2,21]. Estrogen is converted to estradiol, which is involved in key cellular processes in breast cancer[22]. To our knowledge, our study is the first to investigate the interaction between pre-diagnostic smoking and obesity and LN status at diagnosis in breast cancer, and due to evidence supporting a pro-inflammatory and tumorigenic role of these lifestyle factors, we suggest that this be explored further.

This study has a number of strengths including reliable histological information and comprehensive information on baseline characteristics of the cohort. However, information regarding smoking and BMI was self-reported, and completion of this questionnaire may have preceded breast cancer diagnosis by many years. Therefore, the obesity and smoking status of women may have changed between BQ and breast cancer diagnosis. While the multivariable adjusted analyses took into account a number of important confounders, residual confounding is possible. It is important to consider the fact that the BQ was self-reported, and while tumour-specific variables were obtained from medical records, it is possible that some patient characteristics such as BMI, NSAID use, and comorbidity status were inaccurately recorded. The small sample size in this study limited the number of subgroup analyses performed. For example, it would be interesting to investigate the effect of pre-diagnostic smoking and/or obesity on the number of positive nodes at diagnosis. Utada et al. have shown that increasing from two to three positive LNs is associated with poorer patient outcomes in women with breast cancer [23]. In this subset of the PLCO data we did not have access to mortality information, and could not assess the association between smoking and/or obesity on breast cancer mortality.

This study suggests that smoking and/or obesity before breast cancer diagnosis was not associated with lymph node metastasis. However, given the evidence to suggest a role for these lifestyle factors in the development and progression of breast cancer, a larger study, with more detailed information on history of smoking and obesity, is warranted.

## Supporting information

S1 Table Pathologic TNM stage for primary breast cancer from AJCC cancer staging manual 5th edition. (DOCX)
