# Supplementary material for: Associations between obesity, smoking and lymph node status at breast cancer diagnosis in the Prostate, Lung, Colorectal and Ovarian (PLCO) Cancer Screening Trial
Source: PLoS One. 2018 Aug 29;13(8):e0202291. doi: 10.1371/journal.pone.0202291 (PMC6114724; doi:10.1371/journal.pone.0202291)
Supplement: S1 Table — (DOCX) [file pone.0202291.s001.docx]

Supplementary Table 1.

Pathologic TNM Stage for Primary Breast Cancer from AJCC Cancer Staging Manual 5th Edition (Greene *et al*, 2002):

| pN1 | pN1a | Only micro-metastasis (none larger than 0.2cm) |
| --- | --- | --- |
|  | pN1b | Metastasis to lymph node(s), any larger than 0.2cm |
|  | pN1bi | Metastasis in 1-3 lymph nodes, any more than 0.2cm and all less than 2cm in greatest dimension |
|  | pN1bii | Metastasis to 4+ lymph nodes, any more than 0.2cm and all less than 2cm in greatest dimension |
|  | pN1biii | Extension of tumour beyond the capsule of a lymph node metastasis less than 2cm in greatest dimension |
|  | pN1biv | Metastasis to a lymph node 2cm or more in greatest dimension |
| pN2 | pN2 | Metastasis to ipsilateral axillary lymph nodes that are fixed to one another or other structures |
| pN3 | pN3 | Metastasis to ipsilateral internal mammary lymph node(s) |
